# Recursive gene selection based on maximum margin criterion: a comparison with SVM-RFE

**DOI:** 10.1186/1471-2105-7-543

**Published:** 2006-12-25

**Authors:** Satoshi Niijima, Satoru Kuhara

**Affiliations:** 1 Department of Bioinformatics, Graduate School of Systems Life Sciences, Kyushu University, Hakozaki 6-10-1, Higashi-ku, Fukuoka 812-8581, Japan; 2 Faculty of Agriculture, Kyushu University, Hakozaki 6-10-1, Higashi-ku, Fukuoka 812-8581, Japan

## Abstract

**Background:**

In class prediction problems using microarray data, gene selection is essential to improve the prediction accuracy and to identify potential marker genes for a disease. Among numerous existing methods for gene selection, support vector machine-based recursive feature elimination (SVM-RFE) has become one of the leading methods and is being widely used. The SVM-based approach performs gene selection using the weight vector of the hyperplane constructed by the samples on the margin. However, the performance can be easily affected by noise and outliers, when it is applied to noisy, small sample size microarray data.

**Results:**

In this paper, we propose a recursive gene selection method using the discriminant vector of the maximum margin criterion (MMC), which is a variant of classical linear discriminant analysis (LDA). To overcome the computational drawback of classical LDA and the problem of high dimensionality, we present efficient and stable algorithms for MMC-based RFE (MMC-RFE). The MMC-RFE algorithms naturally extend to multi-class cases. The performance of MMC-RFE was extensively compared with that of SVM-RFE using nine cancer microarray datasets, including four multi-class datasets.

**Conclusion:**

Our extensive comparison has demonstrated that for binary-class datasets MMC-RFE tends to show intermediate performance between hard-margin SVM-RFE and SVM-RFE with a properly chosen soft-margin parameter. Notably, MMC-RFE achieves significantly better performance with a smaller number of genes than SVM-RFE for multi-class datasets. The results suggest that MMC-RFE is less sensitive to noise and outliers due to the use of average margin, and thus may be useful for biomarker discovery from noisy data.

## Background

Microarray technology allows us to measure the expression levels of thousands of genes simultaneously. A vast amount of data produced by microarrays pose a great challenge on conventional data mining and machine learning methods, because the number of genes often exceeds tens of thousands, whereas the number of samples is at most a few hundred.

Along with clustering and classification of genes and/or samples, gene selection is an important aspect of microarray data analysis, and has been a central issue in recent years [1,2]. Specifically, gene selection is used to identify genes most relevant to sample classification, for example, those differentiate between normal and cancerous tissue samples. Gene selection plays essential roles in classification tasks. It improves the prediction accuracy of classifiers by using only discriminative genes. It also saves computational costs by reducing dimensionality. More importantly, if it is possible to identify a small subset of biologically relevant genes, it may provide insights into understanding the underlying mechanism of a specific biological phenomenon. Also, such information can be useful for designing less expensive experiments by targeting only a handful of genes.

The most common gene selection approach is so-called gene ranking. It is a univariate approach in the sense that each gene is evaluated individually with respect to a certain criterion that represents class discrimination ability. The criteria often used are e.g., t-statistics, the signal-to-noise (S2N) ratio [3,4] and the between-group to within-group (BW) ratio [5]. Although such gene ranking criteria are simple to use, they ignore correlations or interactions among genes, which may be essential to class discrimination and characterization.

Among existing gene selection methods, support vector machine-based recursive feature elimination (SVM-RFE) [6] has become one of the leading methods and is being widely used. It is a multivariate approach, hence the correlations among genes can be taken into account. Moreover, since the selection is based on an SVM classifier, a subset of genes that yields high classification performance can be identified. Recently, the successful application of SVM-RFE has motivated the development of several SVM-based gene selection methods [7-9]. The SVM-based approach performs gene selection using the weight vector of the hyperplane constructed by the samples on the margin, i.e. support vectors. However, while this property may be crucial for achieving good generalization performance, the effect of using support vectors on gene selection remains unclear, especially when it is applied to noisy, small sample size microarray data. A recent work by Li and Yang [10] implies that only penalizing redundant genes for the samples on the margin may lead to poorer performance.

In this paper, we propose a recursive gene selection method based on the maximum margin criterion (MMC) [11], which is a variant of classical linear discriminant analysis (LDA). Guyon *et al*. [6] compared the performance between SVM-RFE and classical LDA-based RFE (LDA-RFE), and claimed that the use of support vectors is critical in eliminating irrelevant genes. However, the comparison is insufficient in the following respects:

• For computational reasons, LDA-RFE was performed by eliminating half of genes at each iteration, whereas SVM-RFE by eliminating one gene at a time.

• Cross-validation was performed improperly [12].

• The comparison was made only on a single dataset.

The computational drawback of classical LDA limits the use of LDA-RFE for gene selection. This paper presents efficient and stable algorithms for MMC-based RFE (MMC-RFE), which overcomes the singularity problem of classical LDA and the problem of high dimensionality. To validate the effectiveness of MMC-RFE, we extensively compare its performance with that of SVM-RFE using nine cancer microarray datasets.

## Results and discussion

### Datasets

In this study, we used nine public datasets of cancer microarrays. Five of the datasets concern binary-class prediction problems: normal versus tumor for Colon cancer [13] and Prostate cancer [14], ALL versus AML for Leukemia [3], and clinical outcome for Medulloblastoma [15] and Breast cancer [16]. Four of the datasets are on multi-class subtype prediction problems: MLL [17], SRBCT [18], CNS [15], and NCI60 [19]. The details of these datasets are described below:

#### Colon cancer dataset [13]

This Affymetrix high-density oligonucleotide array dataset contains 62 samples from 2 classes of colon-cancer patients: 40 normal healthy samples and 22 tumor samples. The expression profiles of 2000 genes are used. The dataset is publicly available at [20].

#### Prostate cancer dataset [14]

This Affymetrix high-density oligonucleotide array dataset contains 102 samples from 2 classes: 50 normal tissue samples and 52 prostate tumor samples. The expression profiles of 12600 genes are used. The dataset is publicly available at [21].

#### Leukemia dataset [3]

This Affymetrix high-density oligonucleotide array dataset contains 38 samples from 2 classes of leukemia: 27 acute lymphoblastic leukemia (ALL) and 11 acute myeloid leukemia (AML). The expression profiles of 7129 genes are used. The dataset is publicly available at [21]. Other 34 samples consisting of 20 ALL and 14 AML are used as an independent test set as mentioned later.

#### Medulloblastoma dataset [15]

This Affymetrix high-density oligonucleotide array dataset contains 60 samples from 2 classes on patient survival with medulloblastoma: 21 treatment failures and 39 survivors. The expression profiles of 7129 genes are used. The dataset is publicly available at [21].

#### Breast cancer dataset [16]

This cDNA microarray dataset contains 76 samples from 2 classes on five-year metastasis-free survival: 33 poor prognosis and 43 good prognosis. The expression profiles of 4918 genes are used. The dataset is publicly available at [22]. Other 19 samples with 12 poor prognosis and 7 good prognosis are used as an independent test set as mentioned later.

#### MLL dataset [17]

This Affymetrix high-density oligonucleotide array dataset contains 57 samples from 3 classes of leukemia: 20 acute lymphoblastic leukemia (ALL), 17 mixed-lineage leukemia (MLL), 20 acute myelogenous leukemia (AML). The expression profiles of 12582 genes are used. The dataset is publicly available at [21]. Note that a test dataset consisting of 15 samples is not used here.

#### SRBCT dataset [18]

This cDNA microarray dataset contains 63 samples from 4 classes of small round blue-cell tumors of childhood (SRBCT): 23 Ewing family of tumors, 20 rhabdomyosarcoma, 12 neuroblastoma, and 8 non-Hodgkin lymphoma. The expression profiles of 2308 genes are used. The dataset is publicly available at [23]. Note that a test dataset consisting of 20 SRBCT and 5 non-SRBCT samples is also available, but is not used here.

#### CNS dataset [15]

This Affymetrix high-density oligonucleotide array dataset contains 42 samples from 5 different tumors of the central nervous system (CNS): 10 medulloblastomas, 10 malignant gliomas, 10 atypical teratoid/rhabdoid tumors, 8 primitive neuro-ectodermal tumors, and 4 human cerebella. The expression profiles of 7129 genes are used. The dataset is publicly available at [21].

#### NCI60 dataset [19]

This cDNA microarray dataset contains 61 samples from 8 classes of human tumor cell lines: 9 breast, 5 CNS, 7 colon, 8 leukemia, 8 melanoma, 9 non-small cell lung carcinoma, 6 ovarian, and 9 renal tumors. The expression profiles of 3938 genes are used. The dataset is publicly available at [24].

### Preprocessing

For the Prostate cancer, Leukemia, Medulloblastoma, MLL, and CNS datasets, expression values were first thresholded with a floor of 100 (10 for Prostate cancer) and a ceiling of 16000, followed by a base 10 logarithmic transform. Then, each sample was standardized to zero mean and unit variance across genes. For the Colon cancer dataset, after a base 10 logarithmic transform, each sample was standardized. For the Breast cancer dataset, after the filtering of genes following [16], each sample was standardized. For the NCI60 dataset, after filtering genes with missing values, a base 2 logarithmic transform and standardization were applied. For the SRBCT dataset, the expression profiles already preprocessed following [18] were used.

### Gene selection methods for comparison

As a baseline gene selection criterion, we employed the S2N ratio [4] for binary-class problems, and the BW ratio [5] for multi-class problems. Top-ranked genes with the largest ratios were used for classification. We primarily compared two algorithms for MMC-RFE, called uncorrelated MMC-RFE and orthogonal MMC-RFE (see Methods), with SVM-RFE. For the SVM classifier, we used both hard-margin SVM and soft-margin SVM with linear kernel. The effect of using support vectors on gene selection may be directly evaluated by hard-margin SVM, i.e. when setting the soft-margin parameter *C *to infinity. The use of soft-margin SVM can alleviate the influence of noise and outliers to some extent and avoid overfitting of the data, with the trade-off between training errors and the margin. In the experiments, we used a wide range of values for the *C *parameter: *C *= {0.001, 0.01, 0.1, 1, 10, 100, 1000}. The extension of SVM to more than two classes is not obvious. Hence, several approaches have been proposed for multi-class SVMs, of which we employed one-versus-all SVM (OVASVM). Ramaswamy *et al*. [25] showed the effectiveness of the OVASVM approach for gene selection and classification, and Weston *et al*. [8] also applied it to gene selection in multi-class problems. In this study, OVASVM-based RFE was performed in the same way as in [8]. For the implementation of SVM-RFE, we exploited the Spider library for MATLAB, which is publicly available from [26].

### Performance evaluation

We assessed the performance of each gene selection method by repeated random splitting; the samples were partitioned randomly in a class proportional manner into a training set consisting of two-thirds of the whole samples and a test set consisting of the held-out one-third of the samples. To avoid selection bias, gene selection was performed using only the training set, and the classification error rate of the learnt classifier was obtained using the test set. This splitting was repeated 100 times. The error rates averaged over the 100 trials and the corresponding standard error rates are reported.

As a baseline classification method, we employed the nearest mean classifier (NMC), which has been found effective for cancer classification [27]. We combined each gene selection method with NMC. Although the nearest neighbor classifier (NNC) was applied as well, NMC consistently showed favorable performance compared with NNC in the repeated random splitting experiments, and thus the results on NMC are reported here. While the performances of the gene selection methods can be compared fair by using the same classifier, SVM-RFE is often used as an integrated method of gene selection and classification, and MMC-RFE may also perform better when used with the MMC classifier (see Methods). With this view, we further compared the performance between SVM-RFE in combination with the SVM classifier and MMC-RFE with the MMC classifier. For multi-class datasets, the OVASVM classifier was used. 

As suggested by Weston *et al*. [8], to save computational time of RFE, we removed half of the genes until less than 1000, and then a single gene at a time. In this study, we do not address the problem of finding the optimum number of genes that would yield highest classification accuracy. Instead, the number of genes was varied from 1 to 100, and the performances were compared for each number of genes.

### Performance comparison for binary-class datasets

**Figure 1:**
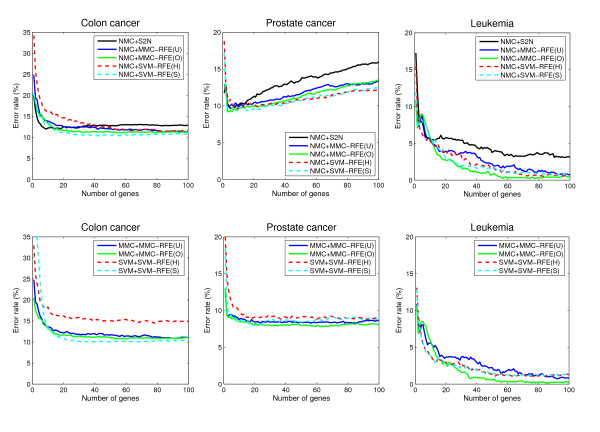
**Performance comparison for binary-class datasets**. The average error rates (%) as a function of the number of genes from 1 to 100, for Colon cancer, Prostate cancer and Leukemia.

**Figure 2:**
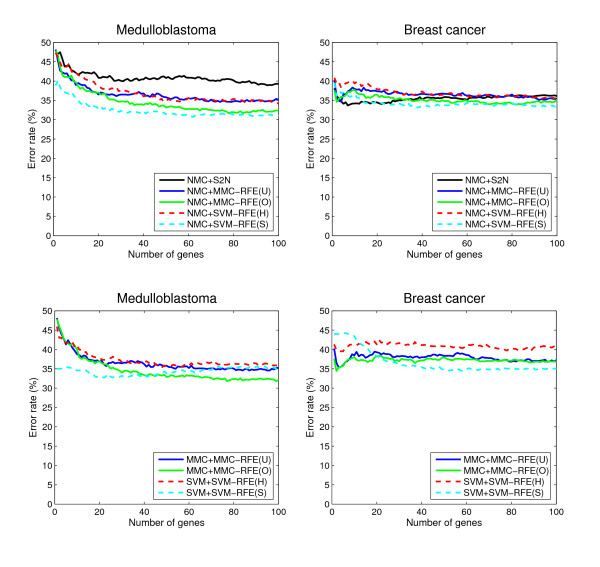
**Performance comparison for binary-class datasets (continued)**. The average error rates (%) as a function of the number of genes from 1 to 100, for Medulloblastoma and Breast cancer.

Tables 1 and 2 show the average error and standard error rates of each combination of classifiers and gene selection criteria for the binary-class datasets: Colon cancer, Prostate cancer, Leukemia, Medulloblastoma, and Breast cancer. Figures 1 and 2 plot the average error rates as a function of the number of genes from 1 to 100. In the tables and figures, MMC-RFE(U), MMC-RFE(O), SVM-RFE(H) and SVM-RFE(S) stand for uncorrelated MMC-RFE, orthogonal MMC-RFE, hard-margin SVM-RFE and soft-margin SVM-RFE, respectively. For SVM-RFE(S), the best result with respect to the *C *parameter is shown. Our observations from these results are as follows:

**Table 1:** Performance comparison for binary-class datasets.

Classifier+Selection criterion	Number of genes				
	
	10	20	30	50	100
**Colon cancer**					

NMC+S2N	12.2 ± 0.6	12.5 ± 0.6	12.3 ± 0.6	12.8 ± 0.6	12.9 ± 0.5

NMC+MMC-RFE(U)	13.9 ± 0.6	12.5 ± 0.6	12.5 ± 0.6	12.1 ± 0.6	11.2 ± 0.5
NMC+MMC-RFE(O)	13.4 ± 0.6	11.7 ± 0.6	11.5 ± 0.6	11.3 ± 0.6	11.2 ± 0.6
NMC+SVM-RFE(H)	16.2 ± 0.7	14.6 ± 0.6	13.7 ± 0.6	12.5 ± 0.6	11.6 ± 0.6
NMC+SVM-RFE(S)	13.3 ± 0.6	11.2 ± 0.6	10.7 ± 0.5	10.5 ± 0.5	10.9 ± 0.5

MMC+MMC-RFE(U)	13.6 ± 0.6	12.1 ± 0.6	11.9 ± 0.6	11.7 ± 0.5	11.0 ± 0.5
MMC+MMC-RFE(O)	13.2 ± 0.6	11.7 ± 0.6	11.5 ± 0.6	11.0 ± 0.6	11.1 ± 0.6
SVM+SVM-RFE(H)	18.3 ± 0.6	16.2 ± 0.7	15.8 ± 0.7	15.0 ± 0.6	15.0 ± 0.6
SVM+SVM-RFE(S)	13.5 ± 0.5	10.7 ± 0.5	10.2 ± 0.5	10.0 ± 0.5	10.5 ± 0.6

**Prostate cancer**					

NMC+S2N	10.1 ± 0.4	11.3 ± 0.5	12.3 ± 0.6	13.6 ± 0.6	16.0 ± 0.7

NMC+MMC-RFE(U)	9.9 ± 0.5	10.4 ± 0.5	10.8 ± 0.5	11.6 ± 0.5	13.4 ± 0.6
NMC+MMC-RFE(O)	9.6 ± 0.5	9.9 ± 0.5	10.3 ± 0.6	11.2 ± 0.6	13.5 ± 0.7
NMC+SVM-RFE(H)	9.6 ± 0.4	10.1 ± 0.5	10.2 ± 0.5	10.8 ± 0.5	12.2 ± 0.6
NMC+SVM-RFE(S)	9.7 ± 0.4	9.6 ± 0.4	10.0 ± 0.5	10.7 ± 0.5	12.4 ± 0.6

MMC+MMC-RFE(U)	8.8 ± 0.4	8.4 ± 0.4	8.4 ± 0.4	8.4 ± 0.4	8.6 ± 0.4
MMC+MMC-RFE(O)	8.5 ± 0.4	8.2 ± 0.4	7.9 ± 0.4	7.9 ± 0.4	8.1 ± 0.5
SVM+SVM-RFE(H)	9.9 ± 0.5	9.1 ± 0.4	9.3 ± 0.4	9.2 ± 0.4	9.1 ± 0.4
SVM+SVM-RFE(S)	8.5 ± 0.4	8.0 ± 0.4	8.5 ± 0.4	8.4 ± 0.4	8.8 ± 0.4

**Leukemia**					

NMC+S2N	5.6 ± 0.7	5.8 ± 0.6	5.4 ± 0.6	3.8 ± 0.5	3.2 ± 0.5

NMC+MMC-RFE(U)	5.7 ± 0.6	3.9 ± 0.5	3.8 ± 0.5	2.2 ± 0.4	0.8 ± 0.2
NMC+MMC-RFE(O)	5.8 ± 0.6	2.8 ± 0.5	1.8 ± 0.4	0.8 ± 0.2	0.4 ± 0.2
NMC+SVM-RFE(H)	5.4 ± 0.6	3.8 ± 0.5	3.4 ± 0.5	1.8 ± 0.4	0.6 ± 0.2
NMC+SVM-RFE(S)	6.0 ± 0.6	3.1 ± 0.4	2.0 ± 0.4	1.5 ± 0.3	0.9 ± 0.3

MMC+MMC-RFE(U)	5.6 ± 0.6	3.7 ± 0.5	3.7 ± 0.5	2.3 ± 0.4	0.8 ± 0.3
MMC+MMC-RFE(O)	5.8 ± 0.6	2.8 ± 0.5	1.6 ± 0.3	0.6 ± 0.2	0.3 ± 0.2
SVM+SVM-RFE(H)	4.1 ± 0.5	3.0 ± 0.4	2.9 ± 0.4	1.3 ± 0.3	1.3 ± 0.3
SVM+SVM-RFE(S)	3.8 ± 0.5	3.1 ± 0.4	2.5 ± 0.4	1.3 ± 0.3	1.3 ± 0.3

The average error and standard error rates (%) for Colon cancer, Prostate cancer and Leukemia, when the number of genes is {10, 20, 30, 50, 100}. SVM-RFE(S) shows the best result with respect to the *C *parameter; NMC+SVM-RFE(S): *C *= 0.01, SVM+SVM-RFE(S): *C *= 0.01 for Colon cancer; NMC+SVM-RFE(S): *C *= 0.01, SVM+SVM-RFE(S): *C *= 0.01 for Prostate cancer; NMC+SVM-RFE(S): *C *= 0.001, SVM+SVM-RFE(S): *C *= 100 for Leukemia.

**Table 2:** Performance comparison for binary-class datasets (continued).

Classifier+Selection criterion	Number of genes				
	
	10	20	30	50	100
**Medulloblastoma**					

NMC+S2N	42.1 ± 1.1	40.9 ± 1.0	40.1 ± 0.9	40.8 ± 1.0	39.3 ± 1.1

NMC+MMC-RFE(U)	39.0 ± 1.0	36.5 ± 1.1	36.5 ± 1.0	35.8 ± 0.9	35.2 ± 1.0
NMC+MMC-RFE(O)	39.7 ± 0.9	37.1 ± 0.9	34.7 ± 0.9	33.2 ± 0.9	32.4 ± 0.9
NMC+SVM-RFE(H)	42.2 ± 1.1	38.5 ± 1.0	37.5 ± 1.0	34.8 ± 0.9	34.3 ± 0.9
NMC+SVM-RFE(S)	35.3 ± 0.9	32.8 ± 0.9	32.3 ± 0.9	31.5 ± 0.9	31.0 ± 0.9

MMC+MMC-RFE(U)	38.8 ± 0.9	36.9 ± 1.0	36.4 ± 1.0	35.8 ± 0.9	35.3 ± 1.0
MMC+MMC-RFE(O)	40.0 ± 0.9	37.0 ± 0.9	34.0 ± 0.9	32.9 ± 0.9	32.2 ± 0.9
SVM+SVM-RFE(H)	41.0 ± 1.0	37.9 ± 0.9	36.8 ± 0.9	35.7 ± 0.9	36.0 ± 0.9
SVM+SVM-RFE(S)	34.6 ± 0.4	32.9 ± 0.6	33.2 ± 0.8	33.9 ± 0.8	34.6 ± 0.8

**Breast cancer**					

NMC+S2N	34.2 ± 0.8	34.5 ± 0.8	35.0 ± 0.8	35.9 ± 0.8	36.1 ± 0.8

NMC+MMC-RFE(U)	38.0 ± 0.8	37.3 ± 0.7	36.8 ± 0.8	36.7 ± 0.7	35.4 ± 0.7
NMC+MMC-RFE(O)	37.7 ± 0.7	36.4 ± 0.7	35.6 ± 0.7	34.8 ± 0.7	35.2 ± 0.7
NMC+SVM-RFE(H)	39.4 ± 0.8	37.8 ± 0.7	36.6 ± 0.8	36.5 ± 0.7	35.6 ± 0.7
NMC+SVM-RFE(S)	36.6 ± 0.9	34.4 ± 0.8	34.1 ± 0.7	33.8 ± 0.7	33.4 ± 0.7

MMC+MMC-RFE(U)	38.5 ± 0.9	39.3 ± 0.7	38.2 ± 0.7	38.4 ± 0.7	37.2 ± 0.8
MMC+MMC-RFE(O)	38.0 ± 0.8	38.2 ± 0.8	37.0 ± 0.7	38.0 ± 0.7	36.9 ± 0.7
SVM+SVM-RFE(H)	41.1 ± 1.0	41.3 ± 0.9	41.7 ± 1.0	40.8 ± 0.8	40.7 ± 0.8
SVM+SVM-RFE(S)	43.4 ± 0.3	38.2 ± 0.6	36.3 ± 0.7	34.8 ± 0.7	35.0 ± 0.7

The average error and standard error rates (%) for Medulloblastoma and Breast cancer, when the number of genes is {10, 20, 30, 50, 100}. SVM-RFE(S) shows the best result with respect to the *C *parameter; NMC+SVM-RFE(S): *C *= 0.001, SVM+SVM-RFE(S): *C *= 0.01 for Medulloblastoma; NMC+SVM-RFE(S): *C *= 0.001, SVM+SVM-RFE(S): *C *= 0.001 for Breast cancer.

• NMC+MMC-RFE(U,O) versus NMC+SVM-RFE(H,S) – Overall, MMC-RFE(U,O) shows intermediate performance between SVM-RFE(H) and SVM-RFE(S) with the best *C *parameter. MMC-RFE(O) is consistently better than MMC-RFE(U), and notably MMC-RFE(O) performs the best for Leukemia. In most cases, however, the difference is not significant and they are quite competitive.

• MMC+MMC-RFE(U,O) versus SVM+SVM-RFE(H,S) – The performance of MMC-RFE(U,O) is improved for Prostate cancer. For the other datasets, the trend is similar to the case of using NMC.

• S2N versus MMC-RFE(U,O), SVM-RFE(H,S) – Both MMC-RFE(U,O) and SVM-RFE(H,S) improve the performance of NMC over S2N for Prostate cancer, Leukemia and Medulloblastoma. Wessels *et al*. [27] have reported that NMC with S2N performs the best among various combinations of gene selection methods and classifiers for Colon cancer and Breast cancer. Consistently with their results, S2N performs better than SVM-RFE(H) for these datasets. However, a significant improvement is achieved for SVM-RFE(S) by setting the *C *parameter to a small value, e.g. 0.001. Huang and Kecman [28] also reported that the finer tuning of the *C *parameter can significantly improve the performance of SVM-RFE.

Guyon *et al*. [6] have drawn a conclusion from their result on the Colon cancer dataset that SVM-RFE performs better than both S2N and LDA-RFE. In their experiment, the *C *parameter was set to 100. However, SVM-RFE(S) with *C *= 100 gives almost the same error rate as SVM-RFE(H) for all the binary-class datasets in our study, and its performance is poorer than that of S2N for Colon cancer, as mentioned previously. There are some reasons that account for this contradiction. First, although Guyon *et al*. [6] used SVM and weighted voting [3] for classification, we have found that for the Colon cancer dataset, SVM with *C *= 100 performs significantly worse than NMC when combined with S2N. As can be seen from Table 1, NMC+SVM-RFE(H) performs even favorably against SVM+SVM-RFE(H). Second, this can be attributed to the selection bias caused by their improper use of cross-validation [12]; they failed to include the gene selection process in the cross-validation. Finally, the performance difference between LDA-RFE and SVM-RFE may be due to the difference in the number of genes eliminated at a time.

Guyon *et al*. [6] also compared the performance between the mean squared error-based RFE (MSE-RFE) and SVM-RFE, and claimed the superiority of SVM-RFE. However, our results suggest that MSE-RFE might also show better performance in some cases. Indeed, this has been implied by the work of Li and Yang [10], which showed that ridge regression-based RFE performed better than SVM-RFE. It should be noted that MSE is closely related to classical LDA and ridge regression [29,30]. MMC-RFE is still advantageous over LDA-RFE and MSE-RFE, because MMC-RFE does not need to compute the inverse of a matrix, which makes MMC-RFE a computationally efficient and stable method.

As our results indicate, the prediction of clinical outcome is generally more difficult than that of tissue or disease types. The error rates of NMC with S2N for the clinical outcome datasets (Medulloblastoma and Breast Cancer) almost coincide with the results presented in [31], which performed a comparative study on outcome prediction using the same validation strategy as our study. The result for Medulloblastoma shows that the prediction performance can be improved by multivariate gene selection methods such as MMC-RFE and SVM-RFE. However, it is at best an error rate of above 30% on average, when using two-thirds of the samples as a training set.

### Performance comparison for multi-class datasets

**Figure 3:**
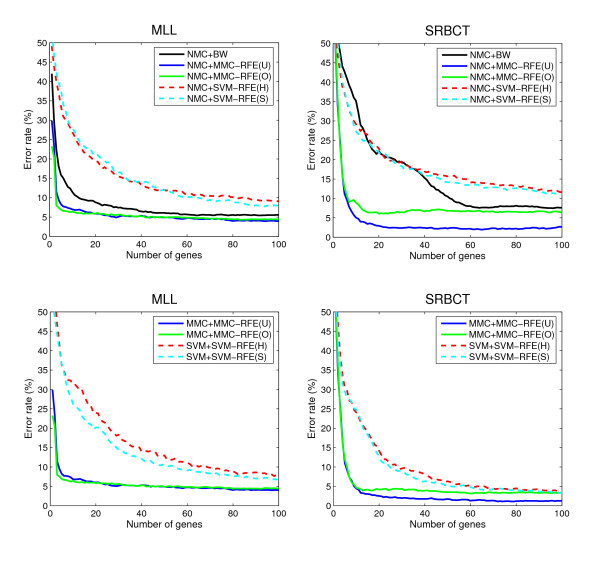
**Performance comparison for multi-class datasets**. The average error rates (%) as a function of the number of genes from 1 to 100, for MLL and SRBCT.

**Figure 4:**
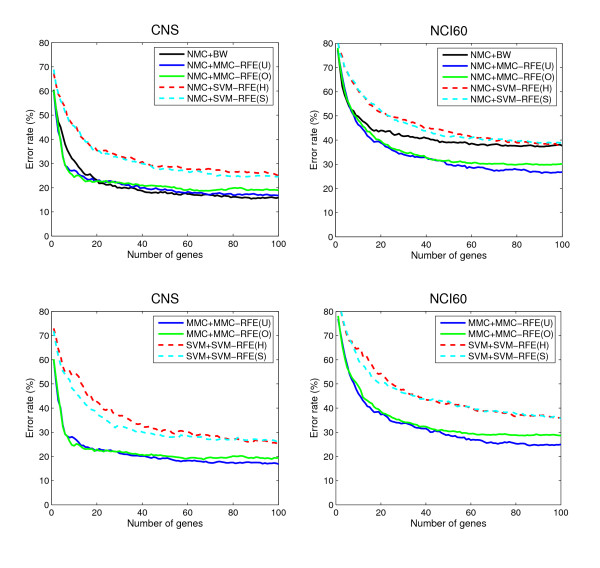
**Performance comparison for multi-class datasets (continued)**. The average error rates (%) as a function of the number of genes from 1 to 100, for CNS and NCI60.

Tables 3 and 4 show the average error and standard error rates of each combination of classifiers and gene selection criteria for the multi-class datasets: MLL, SRBCT, CNS and NCI60. Figures 3 and 4 plot the average error rates as a function of the number of genes from 1 to 100. The OVASVM approach was used here for SVM-RFE. We observe from these results the following:

**Table 3:** Performance comparison for multi-class datasets.

Classifier+Selection criterion	Number of genes				
	
	10	20	30	50	100
**MLL**					

NMC+BW	11.5 ± 0.7	8.8 ± 0.6	7.4 ± 0.5	6.1 ± 0.5	5.6 ± 0.5

NMC+MMC-RFE(U)	7.0 ± 0.6	5.8 ± 0.5	5.1 ± 0.5	4.9 ± 0.5	4.0 ± 0.4
NMC+MMC-RFE(O)	6.4 ± 0.5	5.9 ± 0.5	5.6 ± 0.5	4.9 ± 0.4	4.4 ± 0.4
NMC+SVM-RFE(H)	26.9 ± 1.4	19.3 ± 1.2	15.5 ± 1.1	12.0 ± 0.8	9.1 ± 0.7
NMC+SVM-RFE(S)	28.0 ± 1.3	21.4 ± 1.1	16.6 ± 1.0	11.9 ± 0.8	7.9 ± 0.7

MMC+MMC-RFE(U)	6.8 ± 0.5	6.0 ± 0.5	5.2 ± 0.5	4.9 ± 0.5	4.0 ± 0.4
MMC+MMC-RFE(O)	6.4 ± 0.5	5.8 ± 0.5	5.6 ± 0.5	4.9 ± 0.4	4.5 ± 0.4
SVM+SVM-RFE(H)	31.3 ± 1.5	24.0 ± 1.4	18.3 ± 1.1	12.9 ± 0.8	7.9 ± 0.6
SVM+SVM-RFE(S)	26.2 ± 1.2	20.2 ± 1.1	14.4 ± 1.0	10.6 ± 0.8	6.8 ± 0.6

**SRBCT**					

NMC+BW	35.2 ± 1.4	22.1 ± 0.7	19.3 ± 0.7	10.5 ± 0.7	7.6 ± 0.6

NMC+MMC-RFE(U)	5.0 ± 0.5	3.0 ± 0.4	2.4 ± 0.3	2.2 ± 0.3	2.7 ± 0.3
NMC+MMC-RFE(O)	8.9 ± 0.7	6.0 ± 0.5	6.5 ± 0.5	6.8 ± 0.5	6.4 ± 0.5
NMC+SVM-RFE(H)	29.2 ± 1.2	22.9 ± 1.1	19.5 ± 1.0	15.7 ± 0.9	11.6 ± 0.7
NMC+SVM-RFE(S)	27.2 ± 1.2	21.9 ± 1.2	18.3 ± 1.0	14.2 ± 0.7	11.1 ± 0.8

MMC+MMC-RFE(U)	4.4 ± 0.5	2.5 ± 0.3	2.0 ± 0.3	1.7 ± 0.3	1.3 ± 0.2
MMC+MMC-RFE(O)	4.7 ± 0.5	4.1 ± 0.4	4.4 ± 0.4	3.5 ± 0.4	3.3 ± 0.4
SVM+SVM-RFE(H)	24.0 ± 1.3	14.2 ± 1.0	9.6 ± 0.7	6.3 ± 0.5	3.6 ± 0.4
SVM+SVM-RFE(S)	24.8 ± 1.4	12.7 ± 1.1	8.8 ± 0.8	5.1 ± 0.5	3.4 ± 0.4

The average error and standard error rates (%) for MLL and SRBCT, when the number of genes is {10, 20, 30, 50, 100}. SVM-RFE(S) shows the best result with respect to the *C *parameter; NMC+SVM-RFE(S): *C *= 0.1, SVM+SVM-RFE(S): *C *= 0.1 for MLL; NMC+SVM-RFE(S): *C *= 100, SVM+SVM-RFE(S): *C *= 1000 for SRBCT.

**Table 4:** Performance comparison for multi-class datasets (continued).

Classifier+Selection criterion	Number of genes				
	
	10	20	30	50	100
**CNS**					

NMC+BW	31.1 ± 1.3	23.1 ± 1.2	20.1 ± 1.1	18.3 ± 1.0	15.9 ± 1.0

NMC+MMC-RFE(U)	27.2 ± 1.1	22.8 ± 0.9	21.9 ± 0.9	19.4 ± 0.8	16.8 ± 0.8
NMC+MMC-RFE(O)	24.4 ± 1.0	22.7 ± 0.8	22.1 ± 0.9	20.6 ± 0.9	18.9 ± 0.8
NMC+SVM-RFE(H)	45.6 ± 1.3	35.4 ± 1.0	33.3 ± 1.0	28.8 ± 0.9	24.9 ± 0.8
NMC+SVM-RFE(S)	45.4 ± 1.3	34.9 ± 1.0	32.5 ± 0.9	27.6 ± 0.8	24.6 ± 0.8

MMC+MMC-RFE(U)	27.6 ± 1.1	22.5 ± 0.9	21.3 ± 0.9	19.2 ± 0.8	16.9 ± 0.8
MMC+MMC-RFE(O)	24.4 ± 1.0	22.9 ± 0.8	22.2 ± 0.9	20.2 ± 0.9	19.4 ± 0.8
SVM+SVM-RFE(H)	54.0 ± 1.5	42.6 ± 1.4	36.8 ± 1.3	31.0 ± 0.9	25.2 ± 0.8
SVM+SVM-RFE(S)	47.3 ± 1.2	37.7 ± 1.1	32.6 ± 1.1	28.4 ± 1.0	26.6 ± 0.9

**NCI60**					

NMC+BW	49.8 ± 1.2	44.0 ± 1.0	41.6 ± 1.0	39.1 ± 0.8	37.7 ± 0.7

NMC+MMC-RFE(U)	46.4 ± 0.8	38.9 ± 0.8	34.0 ± 0.9	29.8 ± 0.9	26.8 ± 0.7
NMC+MMC-RFE(O)	48.2 ± 0.9	39.6 ± 0.9	35.0 ± 0.9	31.6 ± 0.8	30.2 ± 0.9
NMC+SVM-RFE(H)	60.6 ± 1.0	51.4 ± 1.0	48.4 ± 1.0	43.4 ± 0.9	38.0 ± 0.8
NMC+SVM-RFE(S)	60.8 ± 1.0	52.2 ± 0.9	47.3 ± 1.0	41.3 ± 0.9	39.0 ± 0.9

MMC+MMC-RFE(U)	46.0 ± 0.9	37.3 ± 0.8	33.7 ± 0.8	29.0 ± 0.9	25.0 ± 0.7
MMC+MMC-RFE(O)	49.0 ± 1.0	38.6 ± 0.9	34.3 ± 0.9	30.4 ± 0.8	28.7 ± 0.9
SVM+SVM-RFE(H)	64.7 ± 1.2	54.3 ± 1.1	47.7 ± 1.0	42.0 ± 0.9	35.9 ± 0.9
SVM+SVM-RFE(S)	59.9 ± 1.1	50.3 ± 1.0	46.2 ± 1.0	42.8 ± 1.1	35.8 ± 0.9

The average error and standard error rates (%) for CNS and NCI60, when the number of genes is {10, 20, 30, 50, 100}. SVM-RFE(S) shows the best result with respect to the *C *parameter; NMC+SVM-RFE(S): *C *= 10, SVM+SVM-RFE(S): *C *= 0.1 for CNS; NMC+SVM-RFE(S): *C *= 100, SVM+SVM-RFE(S): *C *= 0.1 for NCI60.

• NMC+MMC-RFE(U,O) versus NMC+SVM-RFE(H,S) – MMC-RFE(U,O) outperforms SVM-RFE(H,S) for all the datasets; it shows significantly better performance for a smaller number of genes. MMC-RFE(U) appears to be better than MMC-RFE(O) for SRBCT, while they are comparable for the other datasets.

• MMC+MMC-RFE(U,O) versus SVM+SVM-RFE(H,S) – The trend is similar to the case of using NMC. Although the performance of NMC+SVM-RFE(H,S) is improved by SVM+SVM-RFE(H,S) for SRBCT, it is still outperformed by both NMC+MMC-RFE(U,O) and MMC+MMC-RFE(U,O).

• BW versus MMC-RFE(U,O), SVM-RFE(H,S) – MMC-RFE(U,O) shows better performance than BW for three datasets (MLL, SRBCT and NCI60), while performs competitively with BW for CNS. In contrast, SVM-RFE(H,S) performs even worse than BW for these datasets, which suggests that OVASVM may not be suitable for selecting a small number of discriminative genes.

Taken together, our extensive comparison has demonstrated that for binary-class datasets MMC-RFE tends to show intermediate performance between hard-margin SVM-RFE and SVM-RFE with a properly chosen *C *parameter. Notably, MMC-RFE achieves significantly better performance with a smaller number of genes than SVM-RFE for multi-class datasets.

The results on hard-margin SVM-RFE indicate that the use of support vectors is not necessarily effective for achieving better performance in gene selection. Because the SVM-based approach to gene selection uses the weight vector of the hyperplane constructed by the training samples closest to the decision boundary, the performance can be easily affected by noise and possible outliers. As the results on the binary-class datasets show, SVM-RFE can achieve a significant improvement for some of the datasets by setting the *C *parameter to a small value. The soft-margin parameter plays more roles than just handling noisy data; it is effective for linearly inseparable cases and crucial for avoiding overfitting.

In contrast, MMC-RFE uses the discriminant vector obtained by maximizing the average margin, hence less sensitive to noise and outliers. In addition, no parameters need to be tuned. Although MMC-RFE may not be so flexible as soft-margin SVM-RFE, orthogonal MMC-RFE shows comparable performance to SVM-RFE with the best *C *parameter for some cases. Another advantage of MMC-RFE is that it naturally extends to multi-class cases, while the SVM-based approach typically treats them by decomposing the multi-class problems into many binary-class ones, e.g. one-versus-one and one-versus-all strategies. Therefore, MMC-RFE is in particular effective for gene selection in multi-class problems, which has also been validated by the performance on the multi-class datasets.

### Comparison of selected genes

It is clearly of interest to compare the selected genes of MMC-RFE with those of S2N and SVM-RFE. To this end, we conducted additional experiments using independent test sets. The results were obtained for the Prostate cancer, Leukemia, and Breast cancer datasets. Note that the test set for Prostate cancer is from [32], which is available at [33]. It contains 25 normal tissue samples and 9 prostate tumor samples. Gene selection was performed using the whole samples in the previous experiment, and the classification error rate of the learnt classifier was obtained using the independent test set. NNC and NMC were used here for classification, and the number of genes was varied from 1 to 100.

For each dataset, the minimum number of misclassifications as well as the number of genes used are shown in Table 5. Both uncorrelated MMC-RFE and orthogonal MMC-RFE achieve zero misclassifications using a small number of genes for Prostate cancer and Leukemia, although S2N and SVM-RFE also perform comparably well. While S2N and MMC-RFE misclassify three or four test samples for Breast cancer, SVM-RFE yields fewer misclassifications with NMC by using *C *= 0.001.

**Table 5:** Performance comparison for independent test samples.

Dataset	Classifier	# misclassifications (# genes)			
		
		S2N	MMC-RFE(U)	MMC-RFE(O)	SVM-RFE
Prostate cancer	NNC	1 (1)	0 (45)	0 (22)	1 (1)
	NMC	1 (1)	0 (2)	0 (22)	1 (1)
Leukemia	NNC	0 (50)	0 (3)	0 (3)	0 (3)
	NMC	1 (15)	0 (54)	0 (29)	1 (1)
Breast cancer	NNC	4 (19)	3 (91)	3 (85)	4 (2)
	NMC	4 (1)	4 (35)	4 (36)	1 (2)

Minimum number of misclassifications and the number of genes used for Prostate cancer, Leukemia and Breast cancer. The *C *parameter of SVM-RFE was set to 0.01 for Prostate cancer, and to 0.001 for Leukemia and Breast cancer.

Tables 6, 7, 8 list the 10 top-ranked genes of orthogonal MMC-RFE and the corresponding ranks by S2N and SVM-RFE. Note that the lists of uncorrelated MMC-RFE are similar to those of orthogonal MMC-RFE, and hence omitted. For Prostate cancer, 7 genes are included in the list of 16 genes identified by Singh *et al*. [14] (Table 6). Of note, *HPN *(X07732) is ranked the first by all the three gene selection methods. It is known that *hepsin*, a cell surface serine protease, is overexpressed in prostate cancer and has been identified as a potential prostate-cancer biomarker [32,34-36]. *HPN *and *CFD *(M84526) are the two genes that are selected by uncorrelated MMC-RFE and achieve perfect classification with NMC. We can see that some of these genes are also highly ranked by S2N and SVM-RFE. Despite that there are differences in the preprocessing steps and gene selection criteria used, half the genes are included in the lists of the original studies for Leukemia and Breast cancer (Tables 7 and 8); the number of genes identified and used for prediction was 50 for Leukemia [3] and 70 for Breast cancer [16], respectively. It appears that the top-ranked genes of orthogonal MMC-RFE show a larger overlap with those of SVM-RFE than with those of S2N. Indeed, almost all the listed genes belong to the 50 top-ranked genes of SVM-RFE. On the other hand, some of them are assigned small ratio values by S2N. This may be due to the difference in gene selection approaches; MMC-RFE and SVM-RFE are multivariate, whereas S2N is univariate. It is interesting to note that the first rank gene of SVM-RFE for Breast cancer is *PRAME *(NM_006115), which in combination with *TSPYL5 *(AL080059) yields only one misclassification with NMC. The rank of *PRAME *by orthogonal MMC-RFE and S2N is 33 and 107, respectively. Overall, these results show that MMC-RFE can identify a small subset of discriminative genes that is quite consistent with previous studies.

**Table 6:** Comparison of selected genes for Prostate cancer.

Rank	GAN	[14]	Rank		Gene description
				
			S2N	SVM-RFE	
1	X07732	•	1	1	hepsin (transmembrane protease, serine 1) (*HPN*)
2	M30894	•	2	2	TCR gamma alternate reading frame protein (*TARP*)
3	M84526	•	3	89	complement factor D (adipsin) (*CFD*)
4	AL049969	•	4	65	PDZ and LIM domain 5 (*PDLIM5*)
5	X51345		38	5	jun B proto-oncogene (*JUNB*)
6	U21689		68	6	glutathione S-transferase pi (*GSTP1*)
7	M98539	•	297	15	prostaglandin D2 synthase 21kDa (brain) (*PTGDS*)
8	X17206		95	12	ribosomal protein S2 (*RPS2*)
9	D83018	•	6	41	NEL-like 2 (chicken) *(NELL2)*
10	AF065388	•	18	13	tetraspanin 1 (*TSPAN1*)

The 10 top-ranked genes of orthogonal MMC-RFE are listed in order of the rank; GAN: Gene Accession Number. Genes selected by Singh *et al*. [14] are denoted by •. *C *= 0.01 was used for SVM-RFE.

**Table 7:** Comparison of selected genes for Leukemia.

Rank	GAN	[3]	Rank		Gene description
				
			S2N	SVM-RFE	
1	M27891	•	1	2	cystatin C (*CST3*)
2	M28130	•	25	3	interleukin 8 (*IL8*)
3	M84526	•	5	1	D component of complement (adipsin) (*DF*)
4	M19507		131	7	myeloperoxidase (*MPO*)
5	Y00787	•	23	4	interleukin-8 precursor
6	M11722		71	41	deoxynucleotidyltransferase, terminal (*DNTT*)
7	X95735	•	2	11	zyxin (*ZYX*)
8	D88422		3	8	cystatin A (*CSTA*)
9	M27783		15	5	elastase 2, neutrophil (*ELA2*)
10	M96326	•	75	10	azurocidin 1 (*AZU1*)

The 10 top-ranked genes of orthogonal MMC-RFE are listed in order of the rank; GAN: Gene Accession Number. Genes selected by Golub *et al*. [3] are denoted by •. *C *= 0.001 was used for SVM-RFE.

**Table 8:** Comparison of selected genes for Breast cancer.

Rank	GAN	[16]	Rank		Gene description
				
			S2N	SVM-RFE	
1	Contig63649_RC	•	3	40	ESTs
2	AL080059	•	1	2	TSPY-like5 (*TSPYL5*)
3	Contig27312_RC		133	48	collagen, type XXIII, alpha 1 *(COL23A1)*
4	NM_001756		412	35	serpin peptidase inhibitor, clade A (alpha-1 antiproteinase, antitrypsin), member 6 (*SERPINA6*)
5	Contig48328_RC	•	2	4	zinc finger protein 533 (*ZNF533*)
6	NM_001635		69	24	amphiphysin (*AMPH*)
7	NM_006681	•	17	13	neuromedin U (*NMU*)
8	NC_001807		1174	39	Human mitochondrion (*ND1*)
9	NM_000599	•	53	38	insulin-like growth factor binding protein 5 (*IGFBP5*)
10	NM_000518		1387	45	hemoglobin, beta (*HBB*)

The 10 top-ranked genes of orthogonal MMC-RFE are listed in order of the rank; GAN: Gene Accession Number. Genes selected by van't Veer *et al*. [16] are denoted by •. *C *= 0.001 was used for SVM-RFE.

## Conclusion

In this paper, we have proposed a recursive gene selection method based on the MMC, and presented efficient and stable algorithms for MMC-RFE. The performance of MMC-RFE was extensively compared with that of SVM-RFE using nine cancer microarray datasets, including four multi-class datasets. We further compared the top-ranked genes selected by MMC-RFE with those of other gene selection methods, showing the validity of MMC-RFE.

The results suggest that MMC-RFE is less sensitive to noise and outliers due to the use of average margin, while the performance of SVM-RFE can be easily affected by them when applied to noisy, small sample size microarray data. Another advantage of MMC-RFE over SVM-RFE is that MMC-RFE naturally extends to multi-class cases. Furthermore, MMC-RFE does not require the computation of the matrix inversion unlike LDA-RFE and MSE-RFE, and involves no parameters to be tuned.

This study has shown the effectiveness of the MMC for gene selection using microarray data. Our proposed algorithms can also be applied to proteomics and metabolomics datasets, and may be useful for biomarker discovery from such noisy data.

## Methods

### Maximum margin criterion

Linear discriminant analysis (LDA) aims to find a set of projection vectors which maximize the between-class scatter and simultaneously minimize the within-class scatter, thereby achieving maximum discrimination [37].

The between-class scatter matrix ***S***_*b *_and the within-class scatter matrix ***S***_*w *_are defined as

Sb=∑i=1cpi(mi−m)(mi−m)T,Sw=∑i=1cpiSi,
 MathType@MTEF@5@5@+=feaafiart1ev1aaatCvAUfKttLearuWrP9MDH5MBPbIqV92AaeXatLxBI9gBaebbnrfifHhDYfgasaacH8akY=wiFfYdH8Gipec8Eeeu0xXdbba9frFj0=OqFfea0dXdd9vqai=hGuQ8kuc9pgc9s8qqaq=dirpe0xb9q8qiLsFr0=vr0=vr0dc8meaabaqaciaacaGaaeqabaqabeGadaaakeaafaqaaeGadaaabaacbmGae83uam1aaSbaaSqaaiabdkgaIbqabaaakeaacqGH9aqpaeaadaaeWbqaaiabdchaWnaaBaaaleaacqWGPbqAaeqaaOGaeiikaGIae8xBa02aaSbaaSqaaiabdMgaPbqabaGccqGHsislcqWFTbqBcqGGPaqkcqGGOaakcqWFTbqBdaWgaaWcbaGaemyAaKgabeaakiabgkHiTiab=1gaTjabcMcaPmaaCaaaleqabaGaemivaqfaaaqaaiabdMgaPjabg2da9iabigdaXaqaaiabdogaJbqdcqGHris5aOGaeiilaWcabaGae83uam1aaSbaaSqaaiabdEha3bqabaaakeaacqGH9aqpaeaadaaeWbqaaiabdchaWnaaBaaaleaacqWGPbqAaeqaaOGae83uam1aaSbaaSqaaiabdMgaPbqabaaabaGaemyAaKMaeyypa0JaeGymaedabaGaem4yamganiabggHiLdGccqGGSaalaaaaaa@5BBE@

where *c *is the number of classes, ***m***_*i *_and *p*_*i *_are the mean and *a priori *probability of class *i*, ***m ***is the total mean, and ***S***_*i *_is the covariance matrix of class *i*. Then, classical LDA finds the projection vectors ***W ***by maximizing the Fisher criterion

*J *(***W***) = *trace *((***W***^*T *^***S***_*w *_***W***)^-1 ^(***W***^*T *^***S***_*b *_***W***)).     (1)

By solving a generalized eigenvalue problem, the projection vectors ***W ***can be found as the eigenvectors of Sw−1
 MathType@MTEF@5@5@+=feaafiart1ev1aaatCvAUfKttLearuWrP9MDH5MBPbIqV92AaeXatLxBI9gBaebbnrfifHhDYfgasaacH8akY=wiFfYdH8Gipec8Eeeu0xXdbba9frFj0=OqFfea0dXdd9vqai=hGuQ8kuc9pgc9s8qqaq=dirpe0xb9q8qiLsFr0=vr0=vr0dc8meaabaqaciaacaGaaeqabaqabeGadaaakeaaieWacqWFtbWudaqhaaWcbaGaem4DaChabaGaeyOeI0IaeGymaedaaaaa@3164@***S***_*b *_corresponding to the largest eigenvalues. When the sample size is smaller than the dimensionality of samples, however, ***S***_*w *_becomes singular and we cannot compute Sw−1
 MathType@MTEF@5@5@+=feaafiart1ev1aaatCvAUfKttLearuWrP9MDH5MBPbIqV92AaeXatLxBI9gBaebbnrfifHhDYfgasaacH8akY=wiFfYdH8Gipec8Eeeu0xXdbba9frFj0=OqFfea0dXdd9vqai=hGuQ8kuc9pgc9s8qqaq=dirpe0xb9q8qiLsFr0=vr0=vr0dc8meaabaqaciaacaGaaeqabaqabeGadaaakeaaieWacqWFtbWudaqhaaWcbaGaem4DaChabaGaeyOeI0IaeGymaedaaaaa@3164@***S***_*b*_, which is a major drawback of classical LDA.

To overcome the singularity problem, several methods have been proposed e.g. in the field of computer vision, where the number of samples is usually much smaller than the dimensionality. A simple approach is to replace Sw−1
 MathType@MTEF@5@5@+=feaafiart1ev1aaatCvAUfKttLearuWrP9MDH5MBPbIqV92AaeXatLxBI9gBaebbnrfifHhDYfgasaacH8akY=wiFfYdH8Gipec8Eeeu0xXdbba9frFj0=OqFfea0dXdd9vqai=hGuQ8kuc9pgc9s8qqaq=dirpe0xb9q8qiLsFr0=vr0=vr0dc8meaabaqaciaacaGaaeqabaqabeGadaaakeaaieWacqWFtbWudaqhaaWcbaGaem4DaChabaGaeyOeI0IaeGymaedaaaaa@3164@ with the pseudo-inverse matrix $S_{w}^{+}$ . Another approach is to add some constant values to the diagonal elements of ***S***_*w *_as ***S***_*w *_+ *μ****I***, where *μ *> 0 and ***I ***is the identity matrix. However, each of these methods has its own drawbacks and does not scale well to high-dimensional data (see [11] for more details). Recently, Li *et al*. [11] proposed to use the maximum margin criterion (MMC) instead of (1) to find the projection vectors. The MMC is defined as

*J *(***W***) = *trace *(***W***^*T *^(***S***_*b *_- ***S***_*w*_) ***W***)).     (2)

The projection vectors ***W ***= (***w***_1_,..., ***w***_*d*_) which maximize (2) under the constraint that wkT
 MathType@MTEF@5@5@+=feaafiart1ev1aaatCvAUfKttLearuWrP9MDH5MBPbIqV92AaeXatLxBI9gBaebbnrfifHhDYfgasaacH8akY=wiFfYdH8Gipec8Eeeu0xXdbba9frFj0=OqFfea0dXdd9vqai=hGuQ8kuc9pgc9s8qqaq=dirpe0xb9q8qiLsFr0=vr0=vr0dc8meaabaqaciaacaGaaeqabaqabeGadaaakeaaieWacqWF3bWDdaqhaaWcbaGaem4AaSgabaGaemivaqfaaaaa@30E8@***w***_*k *_= 1, *k *= 1,..., *d*, can be found as the eigenvectors of ***S***_*b *_- ***S***_*w *_corresponding to the largest eigenvalues. The advantage of using the MMC is that we need not compute the inverse of ***S***_*w*_, hence the singularity problem can be easily avoided.

It is known that classical LDA can be related to SVM. Shashua [38] has shown that, in binary-class cases, the orientation and location of the hyperplane obtained by SVM is equivalent to the discriminant vector obtained by classical LDA using the samples on the margin. In other words, SVM can be viewed as sparsified LDA. Thus, noting that the MMC is different from classical LDA only in its constraint [11], the major difference between SVM and the MMC consists in that the hyperplane of SVM is constructed only by the training samples closest to the decision boundary, while the discriminant vector of the MMC is constructed so that the average margin computed by all training samples is maximized. They also lead to different problems to solve: a quadratic programming problem for the standard *L*_2 _SVM and an eigenvalue problem for the MMC. Note that for *L*_1 _SVM, it can be reduced to a linear programming problem (see [9] and references therein).

### MMC-RFE algorithms for gene selection

The idea of recursive feature elimination (RFE) [6] is to recursively remove genes using the absolute weights of the discriminant vector or hyperplane, which reflect the significance of the genes for classification. The process starts by training the classifier using all genes. Then, the genes are ranked according to the absolute weights, and those genes with the smallest absolute weights are removed. The classifier is retrained with the remaining genes. This process is repeated until the maximum classification accuracy is obtained or the number of genes reaches a predetermined value. The RFE approach has recently been shown to be effective not only with SVM but also with penalized logistic regression [39] and ridge regression [10].

Here, we propose a recursive gene selection method based on the MMC. The MMC is computationally more efficient and stable than classical LDA, yet it does not scale well to high-dimensional data. When we consider using RFE with the MMC, it is computationally intensive to perform the eigenvalue decomposition (EVD) of the matrix of the gene size in a recursive manner. To overcome the problem of high dimensionality, we first remove the null space of the total scatter matrix via singular value decomposition (SVD) [40], thereby reduce the dimensionality of the data to *n *- 1, where *n *is the number of samples, and then maximize the MMC in the reduced space. Let ***X ***denote the gene expression matrix of size *p *× *n*, where *p *is the number of genes. Then, the total scatter matrix ***S***_*t *_can be expressed as

St=X˜X˜T,
 MathType@MTEF@5@5@+=feaafiart1ev1aaatCvAUfKttLearuWrP9MDH5MBPbIqV92AaeXatLxBI9gBaebbnrfifHhDYfgasaacH8akY=wiFfYdH8Gipec8Eeeu0xXdbba9frFj0=OqFfea0dXdd9vqai=hGuQ8kuc9pgc9s8qqaq=dirpe0xb9q8qiLsFr0=vr0=vr0dc8meaabaqaciaacaGaaeqabaqabeGadaaakeaaieWacqWFtbWudaWgaaWcbaGaemiDaqhabeaakiabg2da9iqb=HfayzaaiaGaf8hwaGLbaGaadaahaaWcbeqaaiabdsfaubaakiabcYcaSaaa@3560@

where

X˜=1n(X−meT),
 MathType@MTEF@5@5@+=feaafiart1ev1aaatCvAUfKttLearuWrP9MDH5MBPbIqV92AaeXatLxBI9gBaebbnrfifHhDYfgasaacH8akY=wiFfYdH8Gipec8Eeeu0xXdbba9frFj0=OqFfea0dXdd9vqai=hGuQ8kuc9pgc9s8qqaq=dirpe0xb9q8qiLsFr0=vr0=vr0dc8meaabaqaciaacaGaaeqabaqabeGadaaakeaaieWacuWFybawgaacaiabg2da9maalaaabaGaeGymaedabaWaaOaaaeaacqWGUbGBaSqabaaaaOGaeiikaGIae8hwaGLaeyOeI0Iae8xBa0Mae8xzau2aaWbaaSqabeaacqWGubavaaGccqGGPaqkcqGGSaalaaa@3A56@

and ***e ***= (1,1,..., 1)^*T *^is an *n*-dimensional vector. Let us assume that *p *> *n *and perform the reduced SVD of X˜
 MathType@MTEF@5@5@+=feaafiart1ev1aaatCvAUfKttLearuWrP9MDH5MBPbIqV92AaeXatLxBI9gBaebbnrfifHhDYfgasaacH8akY=wiFfYdH8Gipec8Eeeu0xXdbba9frFj0=OqFfea0dXdd9vqai=hGuQ8kuc9pgc9s8qqaq=dirpe0xb9q8qiLsFr0=vr0=vr0dc8meaabaqaciaacaGaaeqabaqabeGadaaakeaaieWacuWFybawgaacaaaa@2DFC@ as

X˜=U˜Λ˜V˜T      (3),
 MathType@MTEF@5@5@+=feaafiart1ev1aaatCvAUfKttLearuWrP9MDH5MBPbIqV92AaeXatLxBI9gBaebbnrfifHhDYfgasaacH8akY=wiFfYdH8Gipec8Eeeu0xXdbba9frFj0=OqFfea0dXdd9vqai=hGuQ8kuc9pgc9s8qqaq=dirpe0xb9q8qiLsFr0=vr0=vr0dc8meaabaqaciaacaGaaeqabaqabeGadaaakeaaieWacuWFybawgaacaiabg2da9iqb=vfavzaaiaacceGaf43MdWKbaGaacuWFwbGvgaacamaaCaaaleqabaGaemivaqfaaOGaeiilaWcaaa@3550@

where Λ˜
 MathType@MTEF@5@5@+=feaafiart1ev1aaatCvAUfKttLearuWrP9MDH5MBPbIqV92AaeXatLxBI9gBaebbnrfifHhDYfgasaacH8akY=wiFfYdH8Gipec8Eeeu0xXdbba9frFj0=OqFfea0dXdd9vqai=hGuQ8kuc9pgc9s8qqaq=dirpe0xb9q8qiLsFr0=vr0=vr0dc8meaabaqaciaacaGaaeqabaqabeGadaaakeaaiiqacuWFBoatgaacaaaa@2E35@ = *diag *(λ_1_,..., λ_*n*_) with decreasing non-negative values, and U˜
 MathType@MTEF@5@5@+=feaafiart1ev1aaatCvAUfKttLearuWrP9MDH5MBPbIqV92AaeXatLxBI9gBaebbnrfifHhDYfgasaacH8akY=wiFfYdH8Gipec8Eeeu0xXdbba9frFj0=OqFfea0dXdd9vqai=hGuQ8kuc9pgc9s8qqaq=dirpe0xb9q8qiLsFr0=vr0=vr0dc8meaabaqaciaacaGaaeqabaqabeGadaaakeaaieWacuWFvbqvgaacaaaa@2DF6@ and V˜
 MathType@MTEF@5@5@+=feaafiart1ev1aaatCvAUfKttLearuWrP9MDH5MBPbIqV92AaeXatLxBI9gBaebbnrfifHhDYfgasaacH8akY=wiFfYdH8Gipec8Eeeu0xXdbba9frFj0=OqFfea0dXdd9vqai=hGuQ8kuc9pgc9s8qqaq=dirpe0xb9q8qiLsFr0=vr0=vr0dc8meaabaqaciaacaGaaeqabaqabeGadaaakeaaieWacuWFwbGvgaacaaaa@2DF8@ are *p *× *n *and *n *× *n *orthonormal matrices. Since the rank of ***S***_*t *_is *n *- 1, i.e. λ_*n *_= 0, we can rewrite (3) as

X˜
 MathType@MTEF@5@5@+=feaafiart1ev1aaatCvAUfKttLearuWrP9MDH5MBPbIqV92AaeXatLxBI9gBaebbnrfifHhDYfgasaacH8akY=wiFfYdH8Gipec8Eeeu0xXdbba9frFj0=OqFfea0dXdd9vqai=hGuQ8kuc9pgc9s8qqaq=dirpe0xb9q8qiLsFr0=vr0=vr0dc8meaabaqaciaacaGaaeqabaqabeGadaaakeaaieWacuWFybawgaacaaaa@2DFC@ = ***U*Λ*V***^*T*^,

where **Λ **= *diag *(λ_1_,..., λ_*n *- 1_), and ***U ***and ***V ***are *p *× (*n *- 1) and *n *× (*n *- 1) matrices consisting of the corresponding (*n *- 1) vectors. Thus, we can reduce the dimension by projecting ***X ***onto the (*n *- l)-dimensional space as

***Z ***= **Λ**^-1 ^***U***^*T *^***X***.     (4)

Then, we may maximize the MMC on ***Z***, which is a (*n *- 1) × *n *matrix. Here, we require ***W ***to be orthogonal, i.e. ***W***^*T *^***W ***= ***I***, in the reduced space. Once the discriminant vectors ***W ***of size (*n *- 1) × *d *is obtained, they are projected back onto the original *p*-dimensional space by

W˜
 MathType@MTEF@5@5@+=feaafiart1ev1aaatCvAUfKttLearuWrP9MDH5MBPbIqV92AaeXatLxBI9gBaebbnrfifHhDYfgasaacH8akY=wiFfYdH8Gipec8Eeeu0xXdbba9frFj0=OqFfea0dXdd9vqai=hGuQ8kuc9pgc9s8qqaq=dirpe0xb9q8qiLsFr0=vr0=vr0dc8meaabaqaciaacaGaaeqabaqabeGadaaakeaaieWacuWFxbWvgaacaaaa@2DFA@ = ***U *Λ**^-1 ^***W***,     (5)

where W˜
 MathType@MTEF@5@5@+=feaafiart1ev1aaatCvAUfKttLearuWrP9MDH5MBPbIqV92AaeXatLxBI9gBaebbnrfifHhDYfgasaacH8akY=wiFfYdH8Gipec8Eeeu0xXdbba9frFj0=OqFfea0dXdd9vqai=hGuQ8kuc9pgc9s8qqaq=dirpe0xb9q8qiLsFr0=vr0=vr0dc8meaabaqaciaacaGaaeqabaqabeGadaaakeaaieWacuWFxbWvgaacaaaa@2DFA@ is of size *p *× *d*. Finally, gene selection can be performed using W˜
 MathType@MTEF@5@5@+=feaafiart1ev1aaatCvAUfKttLearuWrP9MDH5MBPbIqV92AaeXatLxBI9gBaebbnrfifHhDYfgasaacH8akY=wiFfYdH8Gipec8Eeeu0xXdbba9frFj0=OqFfea0dXdd9vqai=hGuQ8kuc9pgc9s8qqaq=dirpe0xb9q8qiLsFr0=vr0=vr0dc8meaabaqaciaacaGaaeqabaqabeGadaaakeaaieWacuWFxbWvgaacaaaa@2DFA@. When using (4), we can show that the number of the discriminant vectors that correspond to the positive eigenvalues is at most *c *- 1. Because the eigenvalues reflect the discrimination ability, we use the (*c *- 1) discriminant vectors corresponding to the positive eigenvalues, i.e. *d *is set to *c *- 1, and discard those corresponding to the negative eigenvalues.

Li *et al*. [11] proposed another efficient method to compute the projection vectors of the MMC. It is interesting to note that the MMC is related to uncorrelated LDA (ULDA), and we can find that the Li's method is the same as the ULDA algorithm proposed by Ye [41]. It can be shown that W˜
 MathType@MTEF@5@5@+=feaafiart1ev1aaatCvAUfKttLearuWrP9MDH5MBPbIqV92AaeXatLxBI9gBaebbnrfifHhDYfgasaacH8akY=wiFfYdH8Gipec8Eeeu0xXdbba9frFj0=OqFfea0dXdd9vqai=hGuQ8kuc9pgc9s8qqaq=dirpe0xb9q8qiLsFr0=vr0=vr0dc8meaabaqaciaacaGaaeqabaqabeGadaaakeaaieWacuWFxbWvgaacaaaa@2DFA@ in (5) maximizes the MMC on ***X ***under the constraint that W˜
 MathType@MTEF@5@5@+=feaafiart1ev1aaatCvAUfKttLearuWrP9MDH5MBPbIqV92AaeXatLxBI9gBaebbnrfifHhDYfgasaacH8akY=wiFfYdH8Gipec8Eeeu0xXdbba9frFj0=OqFfea0dXdd9vqai=hGuQ8kuc9pgc9s8qqaq=dirpe0xb9q8qiLsFr0=vr0=vr0dc8meaabaqaciaacaGaaeqabaqabeGadaaakeaaieWacuWFxbWvgaacaaaa@2DFA@^*T *^***S***_*t *_W˜
 MathType@MTEF@5@5@+=feaafiart1ev1aaatCvAUfKttLearuWrP9MDH5MBPbIqV92AaeXatLxBI9gBaebbnrfifHhDYfgasaacH8akY=wiFfYdH8Gipec8Eeeu0xXdbba9frFj0=OqFfea0dXdd9vqai=hGuQ8kuc9pgc9s8qqaq=dirpe0xb9q8qiLsFr0=vr0=vr0dc8meaabaqaciaacaGaaeqabaqabeGadaaakeaaieWacuWFxbWvgaacaaaa@2DFA@ = ***I***, and our method turns out to be equivalent to the ULDA algorithm. Hence, we call the algorithm based on (4) uncorrelated MMC-RFE. 

This study also explores the following projection instead of (4):

***Z ***= ***U***^*T *^***X***.     (6)

After obtaining the discriminant vectors ***W ***by maximizing the MMC on ***Z***, they are projected back onto the original *p*-dimensional space by

W˜
 MathType@MTEF@5@5@+=feaafiart1ev1aaatCvAUfKttLearuWrP9MDH5MBPbIqV92AaeXatLxBI9gBaebbnrfifHhDYfgasaacH8akY=wiFfYdH8Gipec8Eeeu0xXdbba9frFj0=OqFfea0dXdd9vqai=hGuQ8kuc9pgc9s8qqaq=dirpe0xb9q8qiLsFr0=vr0=vr0dc8meaabaqaciaacaGaaeqabaqabeGadaaakeaaieWacuWFxbWvgaacaaaa@2DFA@ = ***U W***.    (7)

Note that no discriminant information is lost in the case of (6) [42]. It can be shown that W˜
 MathType@MTEF@5@5@+=feaafiart1ev1aaatCvAUfKttLearuWrP9MDH5MBPbIqV92AaeXatLxBI9gBaebbnrfifHhDYfgasaacH8akY=wiFfYdH8Gipec8Eeeu0xXdbba9frFj0=OqFfea0dXdd9vqai=hGuQ8kuc9pgc9s8qqaq=dirpe0xb9q8qiLsFr0=vr0=vr0dc8meaabaqaciaacaGaaeqabaqabeGadaaakeaaieWacuWFxbWvgaacaaaa@2DFA@ in (7) maximizes the MMC on ***X ***under the constraint that W˜
 MathType@MTEF@5@5@+=feaafiart1ev1aaatCvAUfKttLearuWrP9MDH5MBPbIqV92AaeXatLxBI9gBaebbnrfifHhDYfgasaacH8akY=wiFfYdH8Gipec8Eeeu0xXdbba9frFj0=OqFfea0dXdd9vqai=hGuQ8kuc9pgc9s8qqaq=dirpe0xb9q8qiLsFr0=vr0=vr0dc8meaabaqaciaacaGaaeqabaqabeGadaaakeaaieWacuWFxbWvgaacaaaa@2DFA@^*T *^W˜
 MathType@MTEF@5@5@+=feaafiart1ev1aaatCvAUfKttLearuWrP9MDH5MBPbIqV92AaeXatLxBI9gBaebbnrfifHhDYfgasaacH8akY=wiFfYdH8Gipec8Eeeu0xXdbba9frFj0=OqFfea0dXdd9vqai=hGuQ8kuc9pgc9s8qqaq=dirpe0xb9q8qiLsFr0=vr0=vr0dc8meaabaqaciaacaGaaeqabaqabeGadaaakeaaieWacuWFxbWvgaacaaaa@2DFA@ = ***I***. We call the algorithm based on (6) orthogonal MMC-RFE. We see that the difference between (4) and (6) results in the different constraints of the MMC on ***X***.

**Figure 5:**
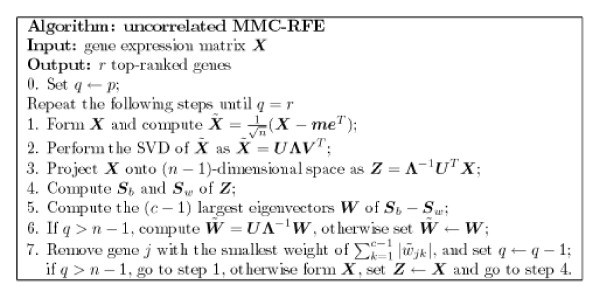
**The uncorrelated MMC-RFE algorithm**.

**Figure 6:**
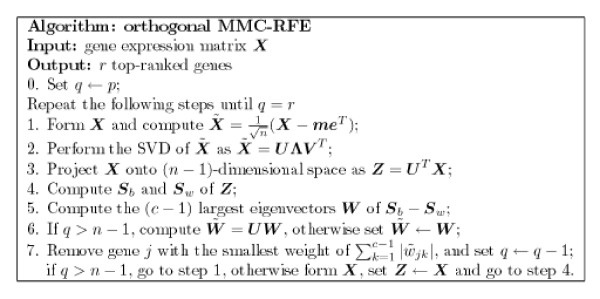
**The orthogonal MMC-RFE algorithm**.

The uncorrelated MMC-RFE and orthogonal MMC-RFE algorithms are summarized in Figures 5 and 6, respectively. They are different in step 3 and step 6. The main computation of both algorithms consists of the SVD of a *p *× *n *matrix at step 2 and the EVD of a (*n *- 1) × (*n *- 1) matrix at step 5. Thus, the algorithms are feasible in the case of high dimensionality and small sample size, i.e. large *p *and small *n*. As is shown, the MMC-RFE algorithms can naturally treat multi-class cases, in which the weight of gene *j *can be defined as the sum of the absolute weights of *c *- 1 discriminant vectors in W˜
 MathType@MTEF@5@5@+=feaafiart1ev1aaatCvAUfKttLearuWrP9MDH5MBPbIqV92AaeXatLxBI9gBaebbnrfifHhDYfgasaacH8akY=wiFfYdH8Gipec8Eeeu0xXdbba9frFj0=OqFfea0dXdd9vqai=hGuQ8kuc9pgc9s8qqaq=dirpe0xb9q8qiLsFr0=vr0=vr0dc8meaabaqaciaacaGaaeqabaqabeGadaaakeaaieWacuWFxbWvgaacaaaa@2DFA@, i.e. ∑k=1c−1|w˜jk|
 MathType@MTEF@5@5@+=feaafiart1ev1aaatCvAUfKttLearuWrP9MDH5MBPbIqV92AaeXatLxBI9gBaebbnrfifHhDYfgasaacH8akY=wiFfYdH8Gipec8Eeeu0xXdbba9frFj0=OqFfea0dXdd9vqai=hGuQ8kuc9pgc9s8qqaq=dirpe0xb9q8qiLsFr0=vr0=vr0dc8meaabaqaciaacaGaaeqabaqabeGadaaakeaadaaeWaqaaiabcYha8jqbdEha3zaaiaWaaSbaaSqaaiabdQgaQjabdUgaRbqabaGccqGG8baFaSqaaiabdUgaRjabg2da9iabigdaXaqaaiabdogaJjabgkHiTiabigdaXaqdcqGHris5aaaa@3CA7@. The maximum of the absolute weights, i.e. max_*k *= 1,..., *c *- 1 _|w˜
 MathType@MTEF@5@5@+=feaafiart1ev1aaatCvAUfKttLearuWrP9MDH5MBPbIqV92AaeXatLxBI9gBaebbnrfifHhDYfgasaacH8akY=wiFfYdH8Gipec8Eeeu0xXdbba9frFj0=OqFfea0dXdd9vqai=hGuQ8kuc9pgc9s8qqaq=dirpe0xb9q8qiLsFr0=vr0=vr0dc8meaabaqaciaacaGaaeqabaqabeGadaaakeaacuWG3bWDgaacaaaa@2E32@_*jk*_|, may also be useful. Note that the uncorrelated MMC-RFE algorithm switches to orthogonal MMC-RFE at *q *= *n *- 1, where *q *is the number of remaining genes during elimination. Hence, our algorithm and the ULDA algorithm may select different genes when *q *≤ *n *- 1.

### MMC classifier

The MMC classifier performs nearest mean classification in the projected space, i.e. the class label *y *of a test sample ***x ***is predicted as

y^=arg⁡min⁡i||WT(x−mi)||2,
 MathType@MTEF@5@5@+=feaafiart1ev1aaatCvAUfKttLearuWrP9MDH5MBPbIqV92AaeXatLxBI9gBaebbnrfifHhDYfgasaacH8akY=wiFfYdH8Gipec8Eeeu0xXdbba9frFj0=OqFfea0dXdd9vqai=hGuQ8kuc9pgc9s8qqaq=dirpe0xb9q8qiLsFr0=vr0=vr0dc8meaabaqaciaacaGaaeqabaqabeGadaaakeaacuWG5bqEgaqcaiabg2da9iGbcggaHjabckhaYjabcEgaNnaaxababaGagiyBa0MaeiyAaKMaeiOBa4galeaacqWGPbqAaeqaaOGaeiiFaWNaeiiFaWhcbmGae83vaC1aaWbaaSqabeaacqWGubavaaGccqGGOaakcqWF4baEcqGHsislcqWFTbqBdaWgaaWcbaGaemyAaKgabeaakiabcMcaPiabcYha8jabcYha8naaCaaaleqabaGaeGOmaidaaOGaeiilaWcaaa@4ABF@

where ***m***_*i *_is the mean of class *i*. Since we perform classification using at most 100 genes in the experiments, the discriminant vectors ***W ***were computed by directly maximizing the MMC under the orthogonality constraint.

### S2N ratio and BW ratio

For each gene *j*, the S2N ratio [4] is defined as

S2N(j)=|μj(1)−μj(2)|σj(1)+σj(2),
 MathType@MTEF@5@5@+=feaafiart1ev1aaatCvAUfKttLearuWrP9MDH5MBPbIqV92AaeXatLxBI9gBaebbnrfifHhDYfgasaacH8akY=wiFfYdH8Gipec8Eeeu0xXdbba9frFj0=OqFfea0dXdd9vqai=hGuQ8kuc9pgc9s8qqaq=dirpe0xb9q8qiLsFr0=vr0=vr0dc8meaabaqaciaacaGaaeqabaqabeGadaaakeaacqqGtbWucqaIYaGmcqqGobGtcqGGOaakcqWGQbGAcqGGPaqkcqGH9aqpdaWcaaqaaiabcYha8HGaciab=X7aTnaaDaaaleaacqWGQbGAaeaacqGGOaakcqaIXaqmcqGGPaqkaaGccqGHsislcqWF8oqBdaqhaaWcbaGaemOAaOgabaGaeiikaGIaeGOmaiJaeiykaKcaaOGaeiiFaWhabaGae83Wdm3aa0baaSqaaiabdQgaQbqaaiabcIcaOiabigdaXiabcMcaPaaakiabgUcaRiab=n8aZnaaDaaaleaacqWGQbGAaeaacqGGOaakcqaIYaGmcqGGPaqkaaaaaOGaeiilaWcaaa@5188@

where μj(1)
 MathType@MTEF@5@5@+=feaafiart1ev1aaatCvAUfKttLearuWrP9MDH5MBPbIqV92AaeXatLxBI9gBaebbnrfifHhDYfgasaacH8akY=wiFfYdH8Gipec8Eeeu0xXdbba9frFj0=OqFfea0dXdd9vqai=hGuQ8kuc9pgc9s8qqaq=dirpe0xb9q8qiLsFr0=vr0=vr0dc8meaabaqaciaacaGaaeqabaqabeGadaaakeaaiiGacqWF8oqBdaqhaaWcbaGaemOAaOgabaGaeiikaGIaeGymaeJaeiykaKcaaaaa@3295@, μj(2)
 MathType@MTEF@5@5@+=feaafiart1ev1aaatCvAUfKttLearuWrP9MDH5MBPbIqV92AaeXatLxBI9gBaebbnrfifHhDYfgasaacH8akY=wiFfYdH8Gipec8Eeeu0xXdbba9frFj0=OqFfea0dXdd9vqai=hGuQ8kuc9pgc9s8qqaq=dirpe0xb9q8qiLsFr0=vr0=vr0dc8meaabaqaciaacaGaaeqabaqabeGadaaakeaaiiGacqWF8oqBdaqhaaWcbaGaemOAaOgabaGaeiikaGIaeGOmaiJaeiykaKcaaaaa@3297@ and σj(1)
 MathType@MTEF@5@5@+=feaafiart1ev1aaatCvAUfKttLearuWrP9MDH5MBPbIqV92AaeXatLxBI9gBaebbnrfifHhDYfgasaacH8akY=wiFfYdH8Gipec8Eeeu0xXdbba9frFj0=OqFfea0dXdd9vqai=hGuQ8kuc9pgc9s8qqaq=dirpe0xb9q8qiLsFr0=vr0=vr0dc8meaabaqaciaacaGaaeqabaqabeGadaaakeaaiiGacqWFdpWCdaqhaaWcbaGaemOAaOgabaGaeiikaGIaeGymaeJaeiykaKcaaaaa@32A2@, σj(2)
 MathType@MTEF@5@5@+=feaafiart1ev1aaatCvAUfKttLearuWrP9MDH5MBPbIqV92AaeXatLxBI9gBaebbnrfifHhDYfgasaacH8akY=wiFfYdH8Gipec8Eeeu0xXdbba9frFj0=OqFfea0dXdd9vqai=hGuQ8kuc9pgc9s8qqaq=dirpe0xb9q8qiLsFr0=vr0=vr0dc8meaabaqaciaacaGaaeqabaqabeGadaaakeaaiiGacqWFdpWCdaqhaaWcbaGaemOAaOgabaGaeiikaGIaeGOmaiJaeiykaKcaaaaa@32A4@ denote the means and standard deviations of two classes, respectively. Top-ranked genes are those with the largest values of S2N(*j*).

The BW ratio [5] can be defined as

$\text{BW}(j)=\frac{\sum_{i}\sum_{k}I(y_{i}=k){\left(\bar{x}._{j}^{\left(k\right)}-\bar{x}._{j}\right)}^{2}}{\sum_{i}\sum_{k}I(y_{i}=k){\left(x_{ij}-\bar{x}._{j}^{\left(k\right)}\right)}^{2}},$

where x¯.j(k)
 MathType@MTEF@5@5@+=feaafiart1ev1aaatCvAUfKttLearuWrP9MDH5MBPbIqV92AaeXatLxBI9gBaebbnrfifHhDYfgasaacH8akY=wiFfYdH8Gipec8Eeeu0xXdbba9frFj0=OqFfea0dXdd9vqai=hGuQ8kuc9pgc9s8qqaq=dirpe0xb9q8qiLsFr0=vr0=vr0dc8meaabaqaciaacaGaaeqabaqabeGadaaakeaacuWG4baEgaqeaiabc6caUmaaDaaaleaacqWGQbGAaeaacqGGOaakcqWGRbWAcqGGPaqkaaaaaa@33BC@ and x¯.j
 MathType@MTEF@5@5@+=feaafiart1ev1aaatCvAUfKttLearuWrP9MDH5MBPbIqV92AaeXatLxBI9gBaebbnrfifHhDYfgasaacH8akY=wiFfYdH8Gipec8Eeeu0xXdbba9frFj0=OqFfea0dXdd9vqai=hGuQ8kuc9pgc9s8qqaq=dirpe0xb9q8qiLsFr0=vr0=vr0dc8meaabaqaciaacaGaaeqabaqabeGadaaakeaacuWG4baEgaqeaiabc6caUmaaBaaaleaacqWGQbGAaeqaaaaa@30AA@ respectively denote the average expression level of gene *j *for class *k *and the overall average expression level of gene *j *across all samples, *y*_*i *_denotes the class of sample *i*, and *I *(·) is the indicator function. Top-ranked genes with the largest values of BW(*j*) are used for classification.

## Authors' contributions

SN designed the experiments, carried out all the analysis, and drafted the manuscript. SK supervised the project. All authors read and approved the final manuscript.
